# Preliminary Results of Preoperative Planning Using 3D Printing and Augmented Reality in Cryotherapy Treatment of Giant Cell Tumor of Bone—CRIO2AR Project

**DOI:** 10.3390/healthcare11192629

**Published:** 2023-09-27

**Authors:** Antonio D’Arienzo, Branimir Scognamiglio, Francesco Rosario Campo, Fabio Cosseddu, Damiano Alfio Ruinato, Edoardo Ipponi, Marina Carbone, Sara Condino, Vincenzo Ferrari, Lorenzo Andreani, Rodolfo Capanna, Paolo Domenico Parchi

**Affiliations:** 1 Department of Orthopedics and Trauma Surgery, University of Pisa, 56124 Pisa, Italy; antonio.darienzo@ao-pisa.toscana.it (A.D.); campofrancescorosario@yahoo.it (F.R.C.); fabiocossedduflore@gmail.com (F.C.); damiano.ruinato@gmail.com (D.A.R.); e.ipponi@studenti.unipi.it (E.I.); lorenzo.andreani.unipi@gmail.com (L.A.); rodolfo.capanna@gmail.com (R.C.); paolo.parchi@unipi.it (P.D.P.); 2 Department of Information Engineering, University of Pisa, 56124 Pisa, Italy; marina.carbone@unipi.it (M.C.); sara.condino@unipi.it (S.C.); vincenzo.ferrari@unipi.it (V.F.); 3 EndoCAS Center, Department of Translational Research and New Technologies in Medicine and Surgery, University of Pisa, 56124 Pisa, Italy

**Keywords:** giant cell tumor of bone, cryotherapy, augmented reality, preoperative planning

## Abstract

Giant Cell Tumor of Bone is a benign tumor with high local aggressive expansion, which, in rare cases, spreads metastasis. Surgical treatment, which often consists of wide curettage to reduce recurrence risk, can lower the quality of life for those affected. Along with aggressive surgery, adjuvant intraoperative techniques have been implemented such as PMMA and cryotherapy. One of the most widely used cryotherapy techniques involves the use of probes to generate ice balls, which have been scientifically shown to have various impacts on the tumor. Although this has been acknowledged, no one has yet tested a way to accurately plan the positioning of cryotherapy probes before surgery, according to the research conducted by the authors. CRIO2AR is a randomized clinical prospective ongoing study by which it will be experimented via preoperative planning of ice probes placement using AR and 3D printing technologies. By studying a single clinical case with these technologies, the surgeon gains better awareness of patient’s anatomy and tumor localization. Preliminary results are shown in the article. The first results are confirming that these technologies are applicable in clinical practice. Secondly, preoperative planning is proving to be reliable, easily replicable, and useful for the surgeon.

## 1. Introduction

Giant Cell Tumor (GCT) of Bone represents about 5% of all primitive bone tumors, and about 20% of the benign ones. Even if mainly considered as a benign tumor, in a percentage of cases, between 1 and 5%, the occurrence of metastasis (mainly lung metastasis or secondary GTC) has been demonstrated. For this reason, a 2020 WHO classification refers to GCT as an intermediate tumor [1,2].

The study of GCT involves performing different instrumental examinations (X-Ray, MRI, scintigraphy) to better characterize the lesion. Nonetheless, there are no examinations that can unequivocally be conclusive for the diagnosis of GCT, which therefore remains confined to histological examination [3].

The classification, developed by Campanacci et al. in 1987, is still used in radiographic staging because of its high correlation with risk recurrence [4].

GCT most commonly affects female subjects between age 20 to 40, and it mainly localizes in the meta-epiphyseal region of long bones, with the knee (as the distal femur, 30% of total GCTs) as the most targeted localization. Other frequent localizations are proximal tibia (28%), distal radius (9%), and distal tibia (6%). Pelvic (2%), sacral (2%), and spinal (3%) are the rarest ones [4]. GCT of bone is characterized by high local aggressiveness and a high rate of recurrence.

Currently, there are no approved medical therapeutic treatments for GTC [3]. Based on lesion localization and extension, main surgical treatments adopted are curettage and resection. In cases when an extended lesion compromises excision surgery, a neoadjuvant treatment with Zoledronic Acid or Denosumab was developed [5,6]. As said before, therefore, surgery is the main solution adopted, which may consist of either wide anatomical resection or curettage and edging of the lesion. Despite aggressive surgical treatments, a recurrence rate of between 27 and 65% of cases has been found. For this reason, adjuvant treatments have been added to surgery over time, including the intraoperative use of phenol, liquid nitrogen, or polymethylmethacrylate (PMMA). When adjuvant surgical techniques are used, recurrence rate is reduced to 12–27% [1]. Among the techniques available in the last three decades, cryotherapy has been introduced during surgery. Studies have shown how to reduce the rate of recurrence when cryotherapy is used [7,8], nevertheless, as of today, its intraoperative use has not been standardized. 

At the II Orthopedic Clinic of Pisa, we have a long history in GCT treatment with the use of cryotherapy as an intraoperative adjuvant [9]. The technology used in our clinic consists of low temperatures produced by argon probes.

The effects of cryotherapy in the adjuvant treatment of neoplastic lesions have been studied for decades. The first studies testing its use in orthopedic surgery belong to Marcove and Miller [10]. Among other observations in their study, they addressed precisely the treatment of TGCs, proposing curettage and cavity filling with liquid nitrogen.

Over the years, research has greatly increased and the biological mechanisms underlying the operation of chemotherapy treatment have been studied and explored.

Cell damage is produced by direct cell freezing and mechanical damage of the cell wall, intracellular dehydration, and ischemia. In addition, regulation of the rate of thawing equally can produce various necrotic effects [11]. Other studies proposed that, beside these effects, there would be also a systemic immunomodulatory response produced because of the sistemical dissemination of tumor antigens [12,13].

Techniques for applying cryotherapy involve the direct use of substances at low temperatures (e.g., liquid nitrogen) or by use of probes within which argon gas flows (this is the method we have adopted). The probes are inserted inside saline solution, poured into the bone cavity, which by freezing produces the desired effects. In specific, probes placement produces ice balls that have different effects on the surrounding tissues depending on the distance between them and, indeed, the probes. It thus appears evident how the placement of the probes affects the final operative result.

Our thinking on the placement of cryoprobes is that they should be placed with cross directions to allow the ice ball to be able to produce its effects in a more even and distributed manner.

Despite the importance of probe placement, to date, no studies proposing preoperative 3D and mixed reality planning can be found in the literature. In this article, we discuss the use of an innovative hybrid simulation and planning platform, based on a desktop application coupled with Augmented Reality (AR) technologies and 3D printing. The feasibility of this platform had been previously tested through the retrospective planning/simulation of the cryotherapy treatment of two surgical cases [14]. In this study, we report the preliminary results of the clinical trial of the system.

## 2. Materials and Methods

Our study is a prospective randomized, controlled, two-arm treatment in which patients treated with adjuvant cryotherapy after standard preoperative planning vs. AR technology and 3D reconstruction planning are compared. Lastly, the two groups will be retrospectively compared with patients treated for GCT at our clinic without the use of cryotherapy.

All three groups will be composed of 5 patients and, for each of them, pre-operative MRI and CT-scans are required. The recruitment phase at the last update counts 6 patients, and for 3 of them, AR pre-operative planning was made (see Table 1).

When a patient affected by GCT comes to our evaluation and, with respect to the inclusion criteria, they are randomly inserted into Group 2 or Group 3. The recruitment phase is still rolling on, so these groups do not count the final 5 patient number yet.

For all the groups, we are evaluating the invasiveness of surgeon procedure, surgeon procedure quality using a LIKERT survey, patient’s quality life using the EORTC QLQ-C30 questionnaire and post-operative pain measured by NRS [15,16,17].

Clinical evaluation of patients and data collection was performed at 2, 6 and 12 months after surgery.

On the other hand, at the same time for the postoperative radiological follow-up, patients were prescribed X-Ray and MRI.

The first step of planning preparation then involves performing a CT scan with slicing of at least 0.6 mm. Starting from the DICOM files, a 3D model is recreated using a semiautomated segmentation software, the EndoCAS Segmentation Pipelin Version Itksnap 1.5.

**Figure 1:**
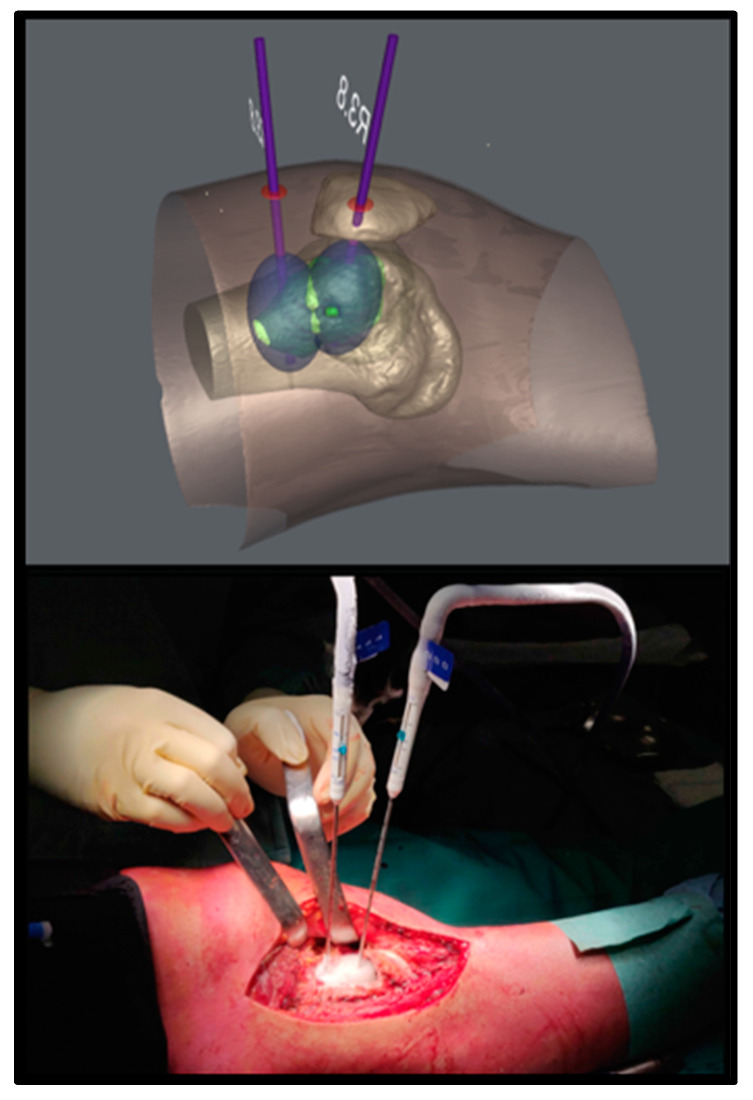
Virtual planning and intraoperative probe positioning of patient n°10.

The desktop application, developed on the cross-platform game engine Unity3D (Unity Technologies Inc., San Francisco, CA, USA) (version 2019.3.15f1), offers the user the following functionalities: 3D visualization and navigation of the patient 3D anatomical models, 3D measurements, selection of different available probes and simulation of their placement. The probes differ in terms of the shape and size of the ice ball generated, and in the needle length and diameter (see Figure 1). Probe models, designed according to the Cryoprobe producer specifications (Endocare Inc., Irvine, CA, USA), allow the simulation of three different isotherms (0°, −20°, −40°): the ice ball sizes were derived from in vitro studies using gelatin-based phantom, and, according to the producer, they approximate (±5 mm) the behavior in soft tissue at 100% gas for 10 min.

Virtual planning can be saved and visualized in a hybrid scenario, which exploits the benefits of 3D printing and the AR functionalities offered by the Microsoft HoloLens Head-Mounted-Display, allowing surgeons to refine the cryotherapy plan in terms of the number, type, and placement of the probes.

In more detail, depending on the particular clinical case, the surgeon selects the anatomical parts to be printed (those whose manipulation can provide haptic feedback useful for a complete understanding of the surgical case) and the anatomical parts that can be visualized in AR. The digital 3D anatomical models are printed in acrylonitrile butadiene styrene (ABS) and a marker, rigidly anchored to the anatomical components, is used for the registration of the virtual content to the real scene (that is, to achieve spatial coherence between the virtual information and the 3D-printed anatomical structures on which it is superimposed).

The clinical application of simulation techniques previously proposed in the literature focused on modeling cryoablation through numerical simulations of heat transfer is limited in that these tools require massively high-computational time [1,7].

The innovative platform proposed in this work does not focus on heat-transfer modeling, but relies on information about ice ball size and isotherms provided by the cryoprobe manufacturer. It allows both visual and tactile inspection of 3D anatomical models in real-time and aims at easing the understanding of the clinical case and improving surgical planning. The foundation of this work is the results of several literature studies that have demonstrated the value of 3D printing and AR for surgical planning and/or simulation [14]. In this work, AR and 3D printing are combined in an innovative hybrid environment, specifically designed to ease cryotherapy procedures, which currently lack objective methods/tools to define probe placement.

## 3. Results

Our study is still ongoing, however, at present, preoperative planning the hybrid platform has been used in three cases. In all three, the localization of the lesion was at the level of the distal femur and, in one of these, it was necessary to proceed with neoadjuvant administration of denosumab because of tumor extension. These three surgical procedures were conducted by three experienced orthopedic surgeons already familiar with the experimental planning and simulation platform. 

Analysis of the responses to the survey postoperatively proposed to the surgeon showed a general improvement in surgery approach. Specifically, the surgeon expressed a score of 5 (extremely agree) to ITEMs 2-4-9-10-12-14-17-18 in all three clinical cases in which the preoperative planning was used. These are ITEMs that questioned, among others, the exact tumor anatomical knowledge and extension and preoperative planning confirmation of probe placement. The placement planning was always confirmed in the operating room. 

Augmented reality has allowed to facilitate probes placement and reduce time spending for doing it (ITEMs 17-18).

There was never any negative impact on surgical scheduling during the course of the study (see Table 2).

As for patient’s result, the EORTC QLQ-C30 questionnaire shows a consistent improvement of ITEMs score as the months since surgery passed by. For patient n°11, the 2-month questionnaire is only available since, at the moment this article has been written, 6 months from surgery has not yet passed.

In the following tables showing the EORTC QLQ-C30 questionnaire results, we have omitted the gastrointestinal domain scores, since no patient has given more than 1 point.

All patients that have completed the 12-month follow-up have not showed lesion recurrence. See results in Table 3 and Table 4.

As mentioned earlier, Group 2 and Group 3 will be compared with the patients previously treated at our clinic, without the use of criotherapy. In view of the fact that this comparison is retrospective, the questionnaire submitted to the patients includes only the equivalent of the 12-month time window. The longest time interval at which the survey was submitted was 18 months. No recurrence was detected in these patients (for patient 3 we consider the surgery performed on the recurrence, following the chronological order for recruiting). For results see Table 5.

## 4. Discussion

In this study, a possible application of a hybrid AR and physical simulator in the field of orthopedic oncologic surgery was evaluated. The main difficulty in conducting surgeries using cryotherapy, by placing probes with argon technology, is to predict the effects of these probes on the surrounding tissues. To this end, planning the cryoprobe placement according to the tumor location/extension plays a fundamental role.

The combined use of a dedicated planning software, 3D printing, and augmented reality has produced remarkable results in this regard. Nowadays, the decision of probe placement relies primarily on the surgeon’s experience and his/her ability to relate probe placement to images from preoperative studies.

In our study, the tools that were made available to surgeons allowed them to complete an important phase of surgery before entering the room, and do it better. The 3D digital phase of probe placement is certainly preliminary and necessary to the AR study of the lesion. In fact, the 3D visualization in mixed reality assumes a much stronger meaning once the surgeon has already decided the number of probes to be placed. Moreover, the decision on the number of freezing cycles to be performed can also be decided at these stages. 

A limitation of the adopted system certainly lies in the absence, as yet, of the development of AR software that would enable probe placement refinement during mixed-reality visualization.

One of the limitations of our study is certainly the small number of patients recruited.

However, considering the type of tumor studied, the purpose of our study and the complexity of the technologies used, we believe that the total number of patients is sufficient.

Moreover, although the total number of patients is less than 15, it seems to well represent the normal distribution of the site localization of the tumor.

Although the conclusion of the study still misses the recruitment of four patients, so far, the successful performance of three surgeries with the technologies under investigation is a highly encouraging result.

## 5. Conclusions

The use of AR technology could enable orthopedic surgeons to better plan the surgical procedure and get better prepared. Thus, establishing in advance the best probe placement could allow selecting prior to surgery the appropriate probe(s) type and estimating the number of cryotherapy cycles that should be taken. For these reasons, we expect that the final results of our clinical study will confirm our preliminary results.

## Figures and Tables

**Table 1:** Patients subdivision into groups.

Patient	GCT Localization	Treatment
GROUP 1—Patient treated without cryotherapy		
1	Proxymal Tibia	Curettage and bone grafting
2	Hand Proxymal Phalanx	Curettage and bone grafting
3	Distal Femur	Curettage and bone grafting
4	Hand Metacarpal	Curettage and bone grafting
5	Distal Femur	Curettage and bone grafting
GROUP 2—Patient treated with cryotherapy and without AR planning		
6	Distal Femur—Recurrence	Curettage and cementation
7	Distal Femur	Curettage and cementation
8	Patella	Curettage and cementation
GROUP 3—Patient treated with cryotherapy and AR planning		
9	Distal Femur	Curettage and cementation
10	Distal Femur	Curettage and cementation
11	Distal Femur	Curettage and bone grafting

**Table 2:** LIKERT survey submitted to surgeons before and after surgery.

Item Score from 1 to 5 (1 = Completely Disagree; 3 = Neutral; 5 = Completely Agree)	Patient and Score										
	1	2	3	4	5	6	7	8	9	10	11
1—It was easy to plan the surgery	3	5	4	5	3	2	2	3	5	5	5
2—I entered the operating room with complete awareness of lesion location and extent	4	5	4	4	3	3	3	4	5	5	5
3—I entered the operating room with a complete awareness of the relationship between the lesion and the surrounding anatomical structures	4	5	5	4	4	4	3	4	5	5	5
4—In the operating room, there were no deviations from what was planned	3	4	5	4	2	3	4	2	5	4	5
5—I am confident of the accuracy of the resection margins	4	5	5	4	4	3	4	3	5	4	4
6—No predictable adverse events occurred in the operating room	5	4	5	5	4	3	3	3	5	5	5
7—I consider the invasiveness of the surgical procedure appropriate to the complexity of the case	4	2	5	2	4	4	4	4	5	5	5
8—I consider the total surgery time adequate compared to the complexity of the case	4	4	5	2	4	4	4	4	5	5	5
Items Administered only in Group 2 and 3 (Patient treated with cryotherapy)											
9—I placed the cryoprobes as planned preoperatively	-	-	-	-	-	4	4	5	5	5	5
10—I am satisfied with the cryoprobes placement	-	-	-	-	-	4	4	4	5	5	5
11—I believe that cryotherapy treatment increased the surgical outcome	-	-	-	-	-	5	5	5	5	5	5
Items Administered only in Group 3 (Preoperative planning with AR and patient treated with cryotherapy)											
12—Planning with augmented reality has been easy	-	-	-	-	-	-	-	-	5	5	5
13—Planning with augmented reality has been helpful	-	-	-	-	-	-	-	-	5	5	5
14—The simulation turned out to be true to reality	-	-	-	-	-	-	-	-	5	5	5
15—Planning with augmented reality allowed me to improve my understanding of the location and extent of the injury	-	-	-	-	-	-	-	-	5	5	5
16—Planning with augmented reality allowed me to improve my understanding of the relationship between the lesion and the surrounding anatomical structures	-	-	-	-	-	-	-	-	5	5	5
17—Planning with augmented reality was helpful to me in defining the placement of cryo-probes	-	-	-	-	-	-	-	-	5	5	5
18—Planning with augmented reality has sped up the placement of cryoprobes	-	-	-	-	-	-	-	-	5	5	5
19—The surgical procedure was smooth and free of any complications	-	-	-	-	-	-	-	-	5	5	5
20—Length of hospitalization has been regular compared to the complexity of the case/intervention	-	-	-	-	-	-	-	-	5	5	4
21—Length of hospitalization has been faster than average considering the complexity of the case/intervention	-	-	-	-	-	-	-	-	3	3	4
22—Length of hospitalization has been slower than average considering the complexity of the case/intervention	-	-	-	-	-	-	-	-	3	3	2

**Table 3:** EORTC QLQ-C30 and NRS scale questionnaire submitted to GROUP 2 patients.

Item Score from 1 to 5 (1 = Completely Disagree; 3 = Neutral; 5 = Completely Agree)	Patient Number											
	6				7				8			
	Months after Surgery											
	0	2	6	12	0	2	6	12	0	2	6	12
1—Do you have trouble doing strenuous activities, like carrying a heavy shopping bag or suitcase?	4	3	2	1	3	2	1	1	5	5	3	2
2—Do you have difficulties taking a long walk?	2	1	1	1	2	2	1	1	5	5	3	2
3—Do you have difficulties taking a short walk outside the house?	2	1	1	1	3	2	1	1	5	4	1	1
4—Do you need to stay in bed or a chair during the day?	2	1	1	1	1	1	1	1	5	5	4	1
5—Do you need help eating, dressing, washing by yourself or using the bathroom?	2	1	1	1	1	1	1	1	2	1	1	1
In the last week:												
6—Were you limited to doing your work or other daily activities?	4	4	4	3	5	5	2	1	5	4	3	2
7—Have you been restricted in pursuing your hobbies or other leisure activities?	4	1	1	1	5	5	1	1	5	4	4	4
8—Were you short of breath?	1	4	2	1	3	3	1	1	4	3	3	2
9—Did you have pain?	4	3	2	1	4	4	1	1	5	3	5	1
10—Did you need to rest?	3	1	1	1	4	4	1	1	5	1	2	1
11—Did you have trouble sleeping?	1	2	1	1	3	3	1	1	5	1	3	1
12—Did you feel weak?	2	1	1	1	3	3	1	1	5	1	1	1
13—Were you tired?	1	4	1	1	1	1	1	1	1	2	3	1
14—Has the pain interfered with your daily activities?	5	1	2	2	3	2	1	1	4	1	1	1
15—Have you had difficulty concentrating on things, like reading a newspaper or watching television?	2	1	1	1	1	1	1	1	1	1	1	1
16—Did you feel tense?	5	5	2	1	3	2	1	1	1	1	1	1
17—Did you get worried?	5	5	1	1	3	1	1	1	1	1	2	1
18—Did you feel irritable?	5	5	1	1	2	1	1	1	1	1	1	1
19—Did you feel depressed?	1	1	1	1	1	1	1	1	1	1	1	1
20—Did you have difficulty remembering things?	2	2	1	1	1	1	1	1	1	1	1	1
21—Has your physical condition or medical treatment interfered with your family life?	2	1	1	1	1	1	2	1	1	1	1	1
22—Has your physical condition or medical treatment interfered with your social activities?	4	3	1	1	1	1	1	1	5	3	1	1
23—Has your physical condition or medical treatment caused you financial difficulties?	3	2	2	1	1	1	1	1	1	1	1	1
24—How would you rate your overall health during the past week?	3	3	4	4	3	2	2	1	3	2	2	1
25—How would you rate your overall quality of life during the past week?	5	4	4	5	4	3	1	1	3	2	3	1
NRS Scale Score	6	3	2	1	6	2	1	1	7	2	1	1

**Table 4:** EORTC QLQ-C30 and NRS scale questionnaire submitted to GROUP 3 patients.

Item Score from 1 to 5 (1 = Completely Disagree; 3 = Neutral; 5 = Completely Agree)	Patient Number											
	9				10				11			
	Months after Surgery											
	0	2	6	12	0	2	6	12	0	2	6	12
1—Do you have trouble doing strenuous activities, like carrying a heavy shopping bag or suitcase?	3	1	1	1	2	1	1		4	1		
2—Do you have difficulties taking a long walk?	5	3	1	1	4	3	1		5	4		
3—Do you have difficulties taking a short walk outside the house?	5	3	1	1	4	3	1		5	4		
4—Do you need to stay in bed or a chair during the day?	5	2	1	1	2	2	1		5	2		
5—Do you need help eating, dressing, washing by yourself or using the bathroom?	3	1	1	1	2	2	1		4	2		
In the last week:												
6—Were you limited to doing your work or other daily activities?	4	3	2	1	3	3	1		5	4		
7—Have you been restricted in pursuing your hobbies or other leisure activities?	5	3	2	1	5	1	2		5	4		
8—Were you short of breath?	1	1	1	1	1	1	1		4	2		
9—Did you have pain?	3	1	1	1	3	2	1		5	3		
10—Did you need to rest?	1	1	1	1	1	1	1		5	1		
11—Did you have trouble sleeping?	1	1	1	1	2	1	1		4	1		
12—Did you feel weak?	1	1	1	1	2	1	1		3	2		
13—Were you tired?	1	1	1	1	3	1	1		1	1		
14—Has the pain interfered with your daily activities?	1	1	1	1	3	2	1		4	3		
15—Have you had difficulty concentrating on things, like reading a newspaper or watching television?	1	1	1	1	1	1	1		2	1		
16—Did you feel tense?	1	1	1	1	1	1	1		5	2		
17—Did you get worried?	5	1	1	1	5	3	1		5	2		
18—Did you feel irritable?	1	1	1	1	2	1	1		5	2		
19—Did you feel depressed?	1	1	1	1	2	1	1		3	2		
20—Did you have difficulty remembering things?	1	1	1	1	1	1	1		1	1		
21—Has your physical condition or medical treatment interfered with your family life?	1	1	1	1	1	1	1		2	1		
22—Has your physical condition or medical treatment interfered with your social activities?	5	3	1	1	5	3	1		5	4		
23—Has your physical condition or medical treatment caused you financial difficulties?	1	1	1	1	3	1	1		3	3		
24—How would you rate your overall health during the past week?	2	4	4	5	2	3	3		2	2		
25—How would you rate your overall quality of life during the past week?	2	3	4	5	2	3	4		2	3		
NRS Scale Score	4	2	1	1	5	4	1		5	1		

**Table 5:** EORTC QLQ-C30 and NRS scale questionnaire submitted to GROUP 1 patients. The survey was conducted when our study started.

Item Score from 1 to 5 (1 = Completely Disagree; 3 = neutral; 5 = Completely Agree)	Patient Number				
	1	2	3	4	5
1—Do you have trouble doing strenuous activities, like carrying a heavy shopping bag or suitcase?	3	1	2	2	1
2—Do you have difficulties taking a long walk?	2	1	3	1	2
3—Do you have difficulties taking a short walk outside the house?	1	1	2	1	1
4—Do you need to stay in bed or a chair during the day?	1	1	2	1	1
5—Do you need help eating, dressing, washing by yourself or using the bathroom?	1	1	1	1	1
In the last week:					
6—Were you limited to doing your work or other daily activities?	2	2	3	1	1
7—Have you been restricted in pursuing your hobbies or other leisure activities?	3	1	4	1	4
8—Were you short of breath?	1	1	1	1	1
9—Did you have pain?	1	1	1	1	1
10—Did you need to rest?	1	1	1	1	1
11—Did you have trouble sleeping?	1	1	1	1	1
12—Did you feel weak?	1	1	1	1	1
13—Were you tired?	1	1	1	1	1
14—Has the pain interfered with your daily activities?	1	1	1	1	1
15—Have you had difficulty concentrating on things, like reading a newspaper or watching television?	1	1	1	1	1
16—Did you feel tense?	1	1	1	1	1
17—Did you get worried?	2	1	1	1	1
18—Did you feel irritable?	1	1	1	1	1
19—Did you feel depressed?	1	1	1	1	1
20—Did you have difficulty remembering things?	1	1	1	1	1
21—Has your physical condition or medical treatment interfered with your family life?	1	1	1	1	1
22—Has your physical condition or medical treatment interfered with your social activities?	2	1	3	1	1
23—Has your physical condition or medical treatment caused you financial difficulties?	1	1	1	1	2
24—How would you rate your overall health during the past week?	4	5	4	1	2
25—How would you rate your overall quality of life during the past week?	5	5	4	5	4
NRS Scale Score	1	1	1	1	2

## Data Availability

The data presented in this study are available on request from the corresponding author.
